# Highly sensitive near-infrared SERS nanoprobes for in vivo imaging using gold-assembled silica nanoparticles with controllable nanogaps

**DOI:** 10.1186/s12951-022-01327-7

**Published:** 2022-03-12

**Authors:** Sungje Bock, Yun-Sik Choi, Minhee Kim, Yewon Yun, Xuan-Hung Pham, Jaehi Kim, Bomi Seong, Wooyeon Kim, Ahla Jo, Kyeong-Min Ham, Sung Gun Lee, Sang Hun Lee, Homan Kang, Hak Soo Choi, Dae Hong Jeong, Hyejin Chang, Dong-Eun Kim, Bong-Hyun Jun

**Affiliations:** 1 Department of Bioscience and Biotechnology, Konkuk University, Seoul, 05029 South Korea; 2 Department of Chemistry Education, Seoul National University, Seoul, 08826 South Korea; 3 Department of Chemical and Biological Engineering, Hanbat National University, Deajeon, 34158 South Korea; 4 Department of Radiology, Gordon Center for Medical Imaging, Massachusetts General Hospital and Harvard Medical School, Boston, MA 02114 USA; 5 Division of Science Education, Kangwon National University, Chuncheon, 24341 South Korea

**Keywords:** Surface-enhanced Raman spectroscopy (SERS), Gold nanoparticle, Nanogap, Hotspot, In vivo imaging

## Abstract

**Background:**

To take advantages, such as multiplex capacity, non-photobleaching property, and high sensitivity, of surface-enhanced Raman scattering (SERS)-based in vivo imaging, development of highly enhanced SERS nanoprobes in near-infrared (NIR) region is needed. A well-controlled morphology and biocompatibility are essential features of NIR SERS nanoprobes. Gold (Au)-assembled nanostructures with controllable nanogaps with highly enhanced SERS signals within multiple hotspots could be a breakthrough.

**Results:**

Au-assembled silica (SiO_2_) nanoparticles (NPs) (SiO_2_@Au@Au NPs) as NIR SERS nanoprobes are synthesized using the seed-mediated growth method. SiO_2_@Au@Au NPs using six different sizes of Au NPs (SiO_2_@Au@Au_50_–SiO_2_@Au@Au_500_) were prepared by controlling the concentration of Au precursor in the growth step. The nanogaps between Au NPs on the SiO_2_ surface could be controlled from 4.16 to 0.98 nm by adjusting the concentration of Au precursor (hence increasing Au NP sizes), which resulted in the formation of effective SERS hotspots. SiO_2_@Au@Au_500_ NPs with a 0.98-nm gap showed a high SERS enhancement factor of approximately 3.8 × 10^6^ under 785-nm photoexcitation. SiO_2_@Au@Au_500_ nanoprobes showed detectable in vivo SERS signals at a concentration of 16 μg/mL in animal tissue specimen at a depth of 7 mm. SiO_2_@Au@Au_500_ NPs with 14 different Raman label compounds exhibited distinct SERS signals upon subcutaneous injection into nude mice.

**Conclusions:**

SiO_2_@Au@Au NPs showed high potential for in vivo applications as multiplex nanoprobes with high SERS sensitivity in the NIR region.

**Graphical Abstract:**

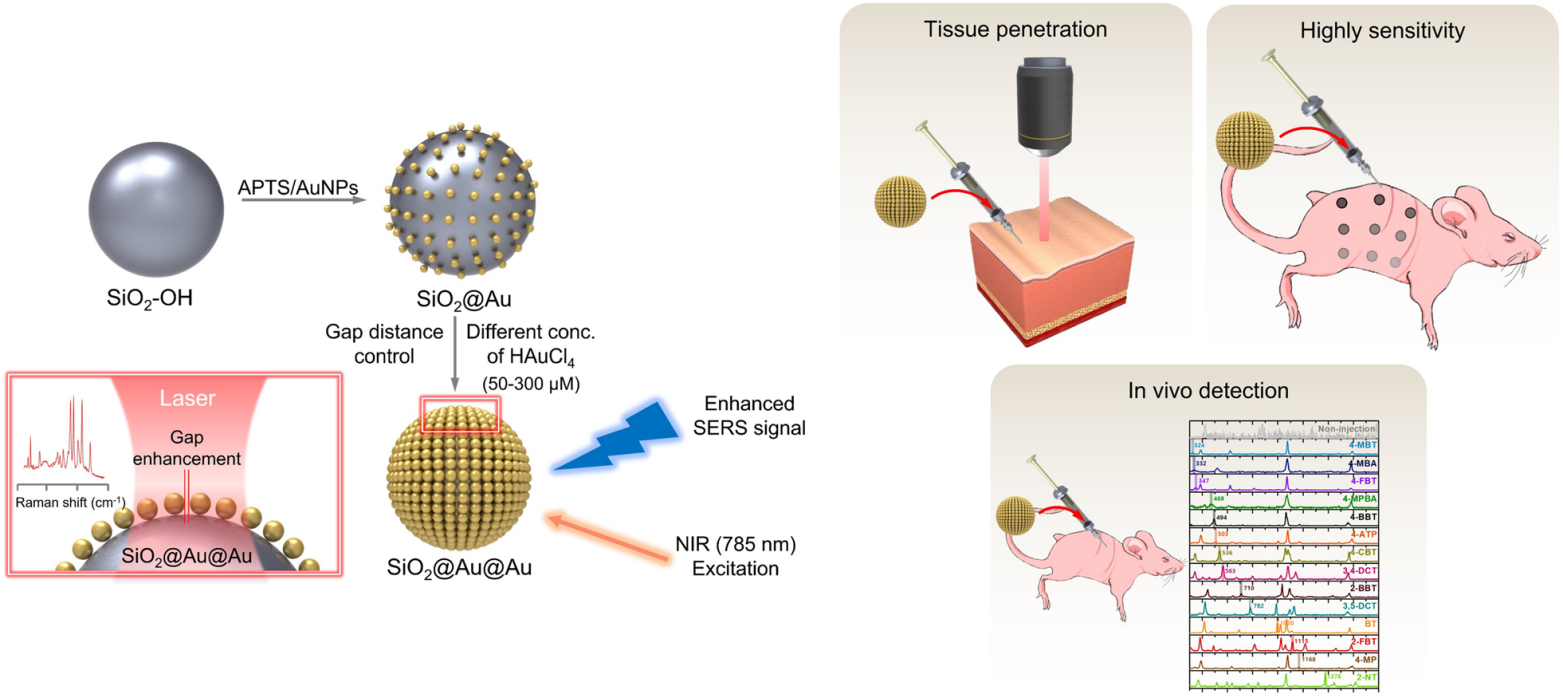

**Supplementary Information:**

The online version contains supplementary material available at 10.1186/s12951-022-01327-7.

## Background

In vivo imaging is a powerful tool for observing the localized effects of drugs as well as biological phenomena in living tissues or organs. However, conventional imaging methods, such as magnetic resonance imaging or molecular imaging based on fluorophores, usually lack multiplex capability [1–3]*.* Moreover, fluorescence imaging suffers from sensitivity issue owing to the autofluorescence of living animal tissues in the presence of visible light. To overcome these problems, in vivo imaging using near-infrared (NIR) light has attracted considerable attention owing to the good penetration ability of NIR radiation into tissues [4–6]. To observe effects of different drugs simultaneously and their multiple tumor-targeting abilities using in vivo imaging techniques, multiplexing capacity is one of the virtues for in vivo imaging probes [7]. However, widely used NIR imaging probes, such as fluorescent dyes and upconversion luminescent nanoparticles (NPs), still exhibit issues of spectral overlap, hindering multiplex imaging [8–10].

Surface-enhanced Raman scattering (SERS) is a powerful tool for biological and chemical analyses and imaging owing to its high sensitivity, multiplexing ability, and selectivity [11–19]. SERS using NIR-active NPs has been applied to multiplex imaging, given the narrow bandwidth (< 2 nm) of Raman signals [20–22]. Among several types of possible SERS NPs, gold NPs (Au NPs) have wide range of bioapplications [23–30] based on the plasmon resonance phenomenon with visible and NIR radiations [31] because of their good biocompatibility and high stability under biological conditions compared to NPs of other metals (for example, silver and copper) [32–34]. However, compared with silver NPs (Ag NPs) that provide better SERS enhancement owing to their strong surface plasmon resonance properties [35], Au NPs still have relatively weak SERS signal enhancement ability.

Many researchers have attempted to build Au nanostructures to embed controllable nanogaps or to be effective as SERS nanoprobes, overcoming the limited SERS enhancement performance of Au NPs [36–38]. Wang et al*.* reported seed-mediated growth method for Au nanostars that exhibited strong absorbance in the NIR region and showed the possibility of imaging and treating cancer cells through photothermal therapy (PTT) [39]. Ding et al*.* reported sea urchin-like, flower-like, meatball-like, and polyhedral Au mesopores of various sizes and shapes [40]. Au NP-assembled nanostructures exhibited strong SERS signals, generating multiple hotspots within the nanogaps between small Au NPs [41]. However, Au NP-assembled nanostructures generated without seed-mediated growth method (usually with a long tedious synthesis process) were heterogenous in shape and had uncontrolled nanogaps.

We recently prepared Au-assembled silica (SiO_2_) NPs by precisely controlling the size of Au NPs on the surface of SiO_2_ NPs [42]. Currently, there are no studies on the relationship between nanogaps within Au NPs and their SERS characteristics, which can be a critical feature for NIR SERS imaging. In this study, Au NP-assembled SiO_2_ NPs (SiO_2_@Au@Au NPs) with small nanogaps were synthesized to develop NIR-active SERS nanoprobes. Various types of nanogaps as SERS hotspots were generated by controlling the degree of Au NP growth on the surface of SiO_2_ NPs. Our SiO_2_@Au@Au NPs showed single-particle level detection sensitivity under 785-nm NIR laser photoexcitation and were applied for in vitro imaging using HCT 116 cell line. To evaluate them as potential SERS nanoprobes, SiO_2_@Au@Au NPs labeled with 4-fluorobenzenethiol (4-FBT) were used to investigate the signal penetration depth in the porcine tissue and the detectable concentration limit upon subcutaneous injection. SiO_2_@Au@Au NPs labeled with 14 types of Raman labeling compounds (RLCs) exhibited distinct Raman spectra and unique bands upon subcutaneous injection. The highly enhanced SERS signals and spectroscopic features of SiO_2_@Au@Au NPs indicate that our NIR nanoprobes have the potential for use in multiplex imaging with various RLCs in vivo.

## Materials and methods

### Materials

Tetraethyl orthosilicate (TEOS), (3-aminopropyl)triethoxysilane (APTS), tetrakis(hydroxymethyl)-phosphonium chloride (THPC), polyvinylpyrrolidone (PVP), gold(III) chloride trihydrate (HAuCl_4_), ascorbic acid (AA), paraformaldehyde, 4-FBT, 2-naphthalenethiol (2-NT), 3,5-dichlorobenzenethiol (3,5-DCT), 4-chlorobenzenethiol (4-CBT), 4-methylbenzenethiol (4-MBT), 4-mercaptophenol (4-MP), 4-bromobenzenethiol (4-BBT), 4-aminothiophenol (4-ATP), 4-mercaptobenzoic acid (4-MBA), 4-mercaptophenyl boronic acid (4-MPBA), benzenethiol (BT), 2-BBT, 3,4-DCT, and 2-FBT were purchased form Sigma-Aldrich (St. Louis, MO, USA). Ethanol (EtOH) and aqueous ammonium hydroxide (NH_4_OH) were purchased from Daejung (Sihung-si, Gyeonggi-do, South Korea). Sodium hydroxide (NaOH) was purchased from Samchun (Pyeongtaek-si, Gyeonggi-do, South Korea). Deionized water (DW) was obtained using a Millipore water purification system (Vivagen, Seongnam-si, Gyeonggi‐do, South Korea). HCT 116 cells were purchased from the American Type Culture Collection (ATCC) (Manassas, VA, USA). Roswell Park Memorial Institute (RPMI) 1640 medium was purchased from Biowest (Riverside, MO, USA). Fetal bovine serum (FBS) was purchased from JCBIO (Seoul, South Korea). Penicillin–streptomycin was purchased from Welgene (Gyeongsan-si, Gyeongsangbuk-do, South Korea). Phosphate-buffered saline (PBS) was purchased from BYLABS (Hanam-si, Gyeonggi-do, South Korea). Sodium dodecyl sulfate (SDS) was purchased from LPS Solution (Daejeon-si, South Korea). Eight-week-old female BALB/c athymic nude mice were purchased from Orient Bio Inc. (Seongnam-si, Gyeonggi-do, Korea).

### Raman spectroscopy measurements

All SERS spectra were obtained using a confocal micro Raman system (XperRF, Nanobase) equipped with an optical microscope (BX41M-LED; Olympus, Tokyo, Japan). The signal was detected using a thermoelectrically cooled (− 60 °C) charge-coupled device detector (Idus 416, Andor Technology). The 532-, 660-, and 785-nm photoexcitation lasers were focused, and the Raman signals except the SERS enhancement factor (EF) calculation data were collected using a × 10 objective lens (0.25 NA, Olympus).

### Preparation of SiO_2_@Au@Au NPs

SiO_2_@Au was synthesized using a previously reported method [42]. Au NPs (3 nm) were prepared using the Turkevich method. Briefly, 1.5 mL of 0.2 M NaOH, 12 μL of THPC, and 1.5 mL of HAuCl_4_ solution (50 mM) were added to 47.5 mL of DW. The mixture was vigorously stirred for 1 h and stored in a refrigerator for at least two days. In addition, 62 μL of APTS and 40 μL of NH_4_OH were added to 1 mL of SiO_2_ NPs (50 mg/mL), and the mixture was stirred overnight at 700 rpm to produce aminated SiO_2_ NPs (SiO_2_-NH_2_ NPs), which were then washed several times with EtOH by centrifugation; subsequently, 10 mL of Au NPs and 200 μL of SiO_2_-NH_2_ NPs (10 mg/mL) were mixed and stirred overnight. SiO_2_@Au NPs were obtained after washing several times with EtOH by centrifugation, which were dispersed in 2 mL of DW containing 2 mg of PVP.

SiO_2_@Au@Au NPs were prepared according to a method described in our previous study [42] with slight modifications. Briefly, SiO_2_@Au@Au NPs were synthesized using the seed-mediated growth method with SiO_2_@Au NP as a seed and Au precursor. To grow Au into SiO_2_@Au seed, 200 μL of SiO_2_@Au NPs (1 mg/mL) was dispersed in 9.8 mL DW containing 10 mg of PVP. This suspension was stirred after adding 20 μL of HAuCl_4_ (10 mM) and treated with 40 μL of AA (10 mM) every 5 min until the desired concentration of Au^3+^ was achieved (50, 100, 200, 300, 400, and 500 μM), which was then washed several times with EtOH by centrifugation to obtain SiO_2_@Au@Au_50_–SiO_2_@Au@Au_500_ NPs.

### Labeling SiO_2_@Au@Au with Raman compounds

An RLC solution (2 mM) was prepared and added to 1 mL of SiO_2_@Au@Au NPs (1 mg/mL). The mixture was vigorously shaken for 1 h at 25 ℃, and thus obtained RLC-conjugated SiO_2_@Au@Au NPs were washed several times with EtOH by centrifugation. Subsequently, Raman-labeled SiO_2_@Au@Au (SiO_2_@Au@Au-RLC) NPs were redispersed in 1 mL of EtOH.

### SERS measurement of SiO_2_@Au@Au-RLC NPs

SiO_2_@Au@Au-RLC suspension (1 mg/mL) was injected into a capillary tube. SERS spectrum of each NP was measured thrice using microscopic Raman system. Measurement was carried out under 532-nm photoexcitation at 1 mW, 660-nm photoexcitation at 1.2 mW, and 785-nm photoexcitation at 2.1 mW laser power using a × 10 objective lens with 5-s acquisition time.

### Calculation of SERS enhancement factor (EF)

Further Raman spectroscopic studies and bioapplication experiments were carried out using SiO_2_@Au@Au_500_, where the SERS EF of SiO_2_@Au@Au_500_-4-FBT NPs under 785-nm photoexcitation was estimated using the following equation: EF = (I_SERS_ × N_normal_)/(I_normal_ × N_SERS_), where I_SERS_ and I_normal_ indicate the intensity of the Raman band from SERS and normal Raman, respectively, and N_normal_ and N_SERS_ are the numbers of 4-FBT molecules in the pure and assembled forms, respectively, on the surface of SiO_2_@Au@Au_500_-4-FBT NPs. The Raman signal intensity was measured for both pure 4-FBT and single-particle level using identical × 100 objective lens (0.90 NA, Olympus) under the following conditions: 0.3 mW laser power and 5-s acquisition time. The 4-FBT peak at 1075 cm^−1^ was used to estimate the EF. I_SERS_ was defined as an average value of the peak intensities of 20 individual particles. The probing volume (18.84 μm^2^) for the normal Raman measurement was approximated using a cylindrical form with a diameter of 2 μm and height of 6 μm. Assuming that 4-FBT molecules form a monolayer on the surface of NPs, N_SERS_ was calculated based on the surface area of NPs (assuming that SiO_2_@Au@Au_500_-4-FBT has a spherical shape, r = 115 nm) and the molecular footprint of 4-FBT (0.383 nm^2^/molecule) [43].

### Cytotoxicity of SiO_2_@Au@Au_500_-4-FBT in HCT 116 cells

HCT 116 cells (human colon cancer cell line) were cultured in RPMI 1640 medium supplemented with 10% heat-inactivated FBS and 1% penicillin/streptomycin at 37 °C in humidified air with 5% CO_2_. Cytotoxicity of NPs was estimated using the crystal violet assay. Cells were seeded in 96-well plates and incubated with different concentrations (0, 1.95, 3.90, 7.81, 15.63, 31.25, and 62.50 mg/mL) of SiO_2_@Au@Au_500_-4-FBT NPs at 37 °C for 24 h. After incubation, the culture medium was removed, and the cells were fixed with 4% paraformaldehyde for 1 h. Then, the cells were washed with DW and air dried. The cells in each well were treated with 100 μL of 0.5% crystal violet solution. After 10 min, the solution was removed and the plates were washed with DW and air dried. Subsequently, the cells were lysed with 1% SDS, and absorbance was measured using VICTOR X3 multilabel plate reader (PerkinElmer, Waltham, MA, USA) at 570 nm.

### SERS imaging of HCT 116 cells

Cells were seeded in a 60-mm dish and incubated with 50 μg/mL SiO_2_@Au@Au_500_-4-FBT NPs at 37 °C for 24 h. After incubation, the culture medium was removed, and the cells were washed thrice with 1 × PBS. Cells were then fixed with 4% paraformaldehyde for 1 h, washed with PBS, and dried at room temperature. Then, the SERS mapping images were obtained by point-by-point mapping (step size: 1 μm) using a × 100 objective lens with a 785-nm excitation source, 0.3-mW laser power, and 1-s acquisition time.

### Depth profile evaluation of SiO_2_@Au@Au SERS signal

To evaluate the depth profile of SiO_2_@Au@Au SERS signal, NPs were injected into the porcine tissue, and the Raman spectra were measured. First, 15 μL of SiO_2_@Au@Au_500_-4-FBT (1 mg/mL) was dispersed in DW and injected into the porcine tissue with a 26-gauge syringe at different depths (1, 3, 5, 7, and 9 mm). SERS signals of NPs inside the tissue were measured immediately after injection using a × 10 objective lens with a 785-nm excitation source, 2.1-mW laser power, and 10-s acquisition time.

### In vivo multiplexing SERS imaging

To conduct multiplexing SERS imaging in nude mice, 14 different types of RLCs (4-MBT, 4-MBA, 4-FBT, 4-MPBA, 4-BBT, 4-ATP, 4-CBT, 3,4-DCT, 2-BBT, 3,5-DCT, BT, 2-FBT, 4-MP, and 2-NT) were conjugated to SiO_2_@Au@Au. After adaptation for one week, the mice were euthanized and subcutaneously injected with 15 μL of SiO_2_@Au@Au_500_-RLC. Diluted SiO_2_@Au@Au_500_-4-FBT (1000, 500, 250, 125, 63, 31, 16, 8, and 4 μg/mL) were injected into another mouse. Each measurement was performed using a × 10 objective lens with a 785-nm excitation source, 2.1-mW laser power, and 10-s acquisition time. Mice were maintained in accordance with the guidelines approved by the Institutional Animal Care and Use Committee (IACUC) of the Konkuk University.

## Results and discussion

### Characterization and SERS properties of SiO_2_@Au@Au NPs

**Fig. 1:**
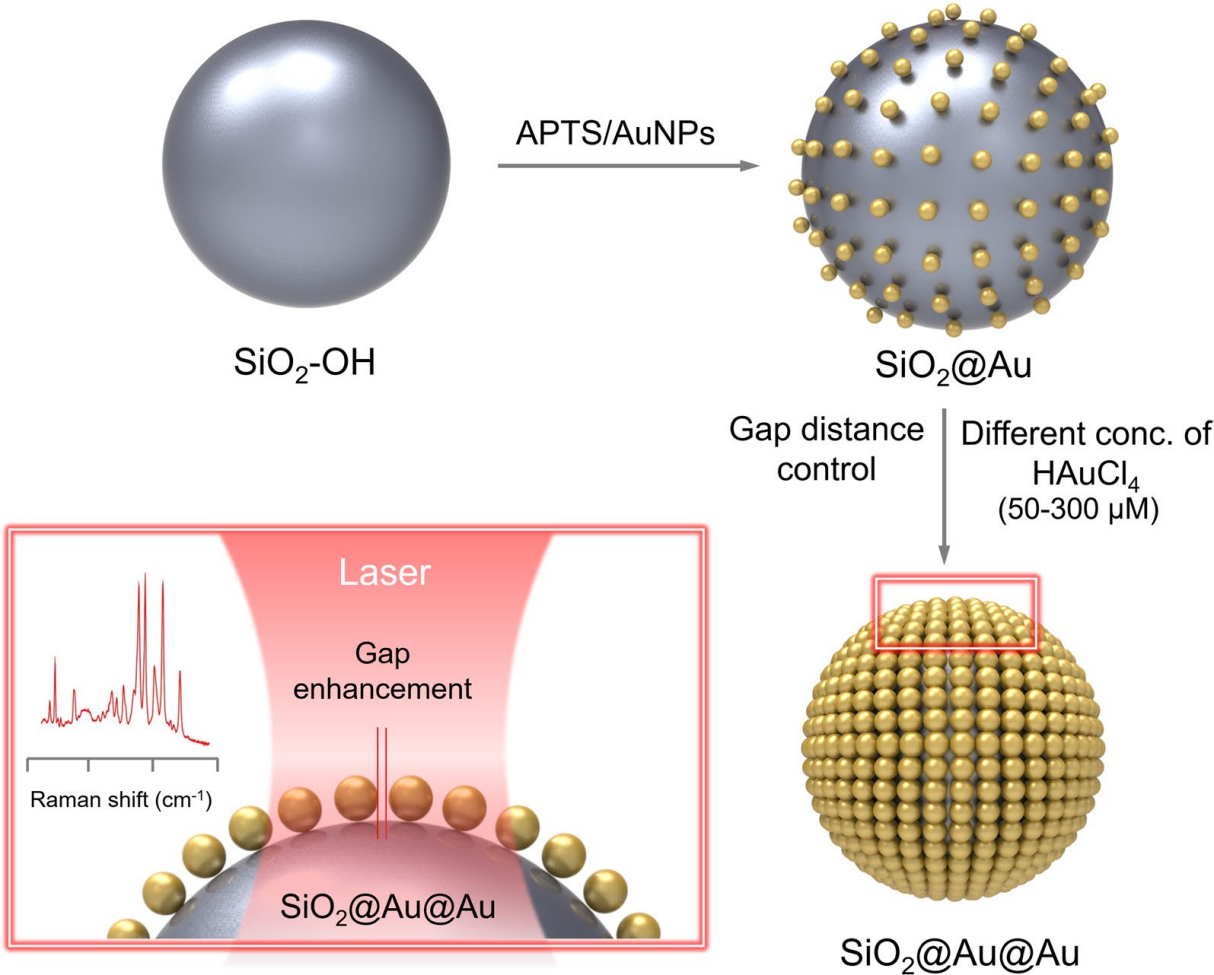
Schematic illustration of the preparation process of SiO_2_@Au@Au using SiO_2_@Au as a seed

**Fig. 2:**
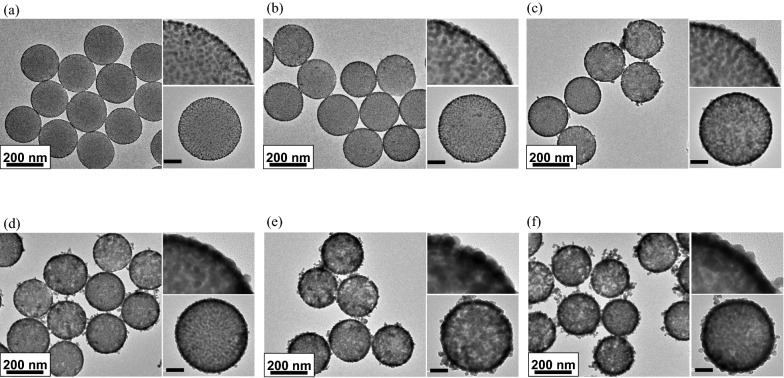
Morphology analysis of SiO_2_@Au@Au containing various concentrations of gold(III) chloride hydrate by transmission electron microscopy. SiO_2_@Au@Au synthesized using (**a**) 50 μM, (**b**) 100 μM, (**c**) 200 μM, (**d**) 300 μM, (**e**) 400 μM, and (**f**) 500 μM gold(III) chloride hydrate. Each scale bar of inset images is 50 nm. Overall size and nanogap between Au NPs were controlled by gold(III) chloride hydrate concentration

SiO_2_@Au@Au NPs were prepared using the method described in our previous study, with modifications [42]. Briefly, SiO_2_@Au@Au was prepared by introducing Au NPs into SiO_2_ to facilitate the growth of Au (Fig. 1). SiO_2_ NPs were prepared according to the Stöber method (Additional file 1: Fig. S1). Subsequently, Au NPs were introduced into SiO_2_ following treatment with APTS. SiO_2_@Au was used as a seed in the seed-mediated growth method (Additional file 1: Fig. S2). SiO_2_@Au seed (195.30 ± 13.16 nm) contained several Au NPs of very small size (3 nm) attached to the SiO_2_ NP surface. It is imperative to control the size of Au NPs on the SiO_2_ core to achieve a gap-enhanced SERS efficacy to create a strong local field between the Au NP gaps. In this regard, SiO_2_@Au@Au NPs were fabricated using SiO_2_@Au NP as a seed with varying concentrations of Au precursor (50, 100, 200, 300, 400, and 500 μM). After application of the growth method, the six prepared SiO_2_@Au@Au NPs were 212.80 ± 7.35, 213.54 ± 7.14, 215.81 ± 8.30, 219.56 ± 9.36, 229.47 ± 9.85, and 229.48 ± 7.27 nm in size, corresponding to Au precursor concentrations of 50, 100, 200, 300, 400, and 500 μM, respectively (Fig. 2). With addition of higher concentration of Au precursor, the overall size of the NPs increased owing to the growth of Au NPs. The maximum concentration of Au precursor was 500 μM to prevent formation of merged structures (loss of particle morphology feature of Au NP) and seedings exclude Si NPs (Additional file 1: Fig. S3). The seed-mediated growth method allowed dense packing of Au NPs on the SiO_2_ core surface, in contrast to the direct attachment of large-sized Au NPs on the SiO_2_ core (Additional file 1: Fig. S4).

**Fig. 3:**
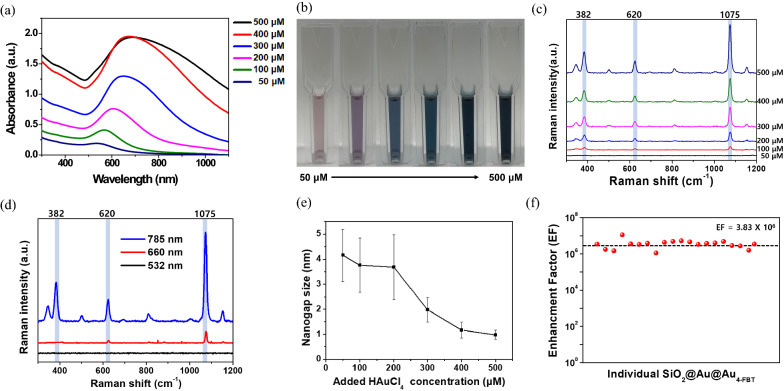
UV/Vis absorbance spectra and Raman spectroscopic characterization of SiO_2_@Au@Au. **a** UV/Vis absorbance spectra of SiO_2_@Au@Au with various concentrations of gold(III) chloride hydrate and (**b**) different optical colors of each SiO_2_@Au@Au. **c** Raman intensities of SiO_2_@Au@Au with various concentrations of gold(III) chloride hydrate captured using a 785-nm laser. (**d**) Raman intensities of SiO_2_@Au@Au_500_-4-FBT using blue visible light (532 nm), red visible light (660 nm), and near-infrared (NIR) light (785 nm) as photoexcitation sources. **e** Nanogap sizes of SiO_2_@Au@Au NPs. **f** Calculated enhancement factor (EF) of single SiO_2_@Au@Au_500_-4-FBT under NIR light based on the SERS intensity of 1075 cm^−1^

Figure 3a shows the absorbance of each prepared SiO_2_@Au@Au NP. The absorbance increased at all wavelengths with increase in Au precursor concentration, particularly in the NIR region. In addition, the maximum absorption wavelength (λ_max_) showed a red-shift with an increase in the concentration of Au precursor. This phenomenon is attributed to strong plasmonic coupling caused by the growth of Au NPs on the SiO_2_ NP surfaces. As the absorbance changed, the color of the NPs dispersed in the solvent (EtOH) changed from light pink to dark blue (Fig. 3b).

To investigate the SERS characteristics of SiO_2_@Au@Au, SERS spectra of the six SiO_2_@Au@Au NPs after treatment with 4-FBT were measured using three different laser lines (532, 660, and 785 nm) (Fig. 3c; Additional file 1: Fig. S4). Raman signals were not detectable for any of the six SiO_2_@Au@Au-4-FBT NPs at 532 nm (Additional file 1: Fig. S4a). This was due to the relatively weak plasmonic resonances of the six SiO_2_@Au@Au NPs irradiated with light at a wavelength of 532 nm. SERS spectra obtained using a 660-nm laser revealed distinct bands for SiO_2_@Au@Au NPs treated with 200, 300, 400, and 500 μM Au precursor (Additional file 1: Fig. S4b). SERS signals measured using a 785-nm laser were stronger than those obtained using a 532-nm laser and 660-nm laser except the SiO_2_@Au@Au NPs treated with 50 μM Au precursor, for which no signal was detected (Fig. 3c; Additional file 1: Fig. S5). A comparison between 4-FBT SERS signal of SiO_2_@Au@Au_500_ at 1075 cm^−1^ peak showed that the Raman intensity with 785-nm photoexcitation was 7.7 times higher than that measured using 660-nm laser (Fig. 3d).

SERS spectra of SiO_2_@Au@Au NPs captured using 660-nm and 785-nm lasers showed enhanced Raman signals with an increase in the number of Au NPs on the SiO_2_ surface. This could be attributed not only to the stronger absorbance but also to the narrower nanogap between Au NPs, leading to a highly amplified SERS signal. Transmission electron microscopy (TEM) images (Fig. 2) show that the nanogaps between Au NPs on the SiO_2_ core gradually decreased as the concentration of Au NPs increased. The nanogap sizes were measured to be 4.16 ± 1.04, 3.76 ± 1.09, 3.68 ± 1.29, 1.98 ± 0.50, 1.17 ± 0.32, and 0.98 ± 0.19 nm for NPs treated with 50, 100, 200, 300, 400, and 500 μM Au precursor, respectively (Fig. 3e). The strongest Raman signal of SiO_2_@Au@Au NPs with 500 μM Au precursor during Au seed growth might be because the electromagnetic field was concentrated in 1-nm nanogaps. The seed-mediated growth method for SiO_2_@Au@Au NPs was validated as a powerful strategy to precisely control the nanogap size and maximize the SERS enhancement.

Considering that SiO_2_@Au@Au_500_ NPs have a higher absorbance in the NIR region and show the strongest Raman enhancement ability among all prepared NPs, the following experiments were performed using SiO_2_@Au@Au_500_. Using 4-FBT molecule as an RLC, the SERS spectra of 20 single particles of SiO_2_@Au@Au_500_-4-FBT were measured, and the average EF value was estimated to be 3.8 × 10^6^ with good uniformity (3.45% relative standard deviation on log scale) (Fig. 3f). Compared to other noble metal-based NPs, the assembled structure had a lower EF value owing to the large surface area for RLC binding. However, the higher intensity and signal uniformity of each nanocomposite could be an advantageous feature of the assembled structures [44]. SiO_2_@Au@Au_500_ exhibited higher EF values than those reported for other noble metal-assembled NPs (Table 1). Although the EF value is smaller than that of bumpy silver nanoshells, Au-assembled SiO_2_ NPs are more stable than Ag-based NPs under biological conditions [45].

**Table 1:** Comparison of enhancement factor of different metal-assembled nanoparticles

Composition	Nanoparticle (NP)	Enhancement factor (EF)	References
Silver (Ag)	Silica-encapsulated Ag-SiO_2_ NP	1 × 10^5^	[47]
	Bumpy silver nanoshell	2.2 × 10^7^	[45]
Gold (Au)	Au/Ag hollow shell-assembled silica nanosphere	2.8 × 10^5^	[48]
	Au-assembled silica NP	3.8 × 10^6^	Current study

### SERS imaging of HCT 116 cancer cell with SiO_2_@Au@Au_500_-4-FBT

Before using SiO_2_@Au@Au for in vitro applications, a cytotoxicity test was conducted using HCT 116 cell line. SiO_2_@Au@Au_500_-4-FBT NPs were prepared at a concentration of 62.5 μg/mL (26.38 × 10^8^ particles/mL) and serially diluted for the cytotoxicity test (Additional file 1: Fig. S6). Cell viability was more than 90% at all concentrations of SiO_2_@Au@Au NPs within 24 h. Moreover, biocompatibility of SiO_2_@Au@Au_500_-4-FBT NPs at a concentration of 62.5 μg/mL or lower was confirmed.

To obtain images of HCT 116 cancer cells through SERS, the cells were incubated with SiO_2_@Au@Au_500_-4-FBT NPs for 24 h. NPs were either attached to the cell surface or entered the cell, whereas the remaining NPs were washed out. Additional file 1: Figure S7a shows the SERS mapping image at 1075 cm^−1^. The overlay image of HCT 116 cells and the adsorbed NPs showed that SiO_2_@Au@Au_500_-4-FBT NPs were attached to the edge of the cell. We compared the Raman intensity at different locations on the cell and observed no Raman signal outside the cell (i), weak Raman signal at the cell surface (ii), and extremely strong Raman signal inside the cell (iii) (Additional file 1: Fig. S7b). This observation confirmed the possibility of SERS imaging of cancer cells using SiO_2_@Au@Au NPs.

### Sensitivity of SERS signal of SiO_2_@Au@Au_500_-4-FBT

**Fig. 4:**
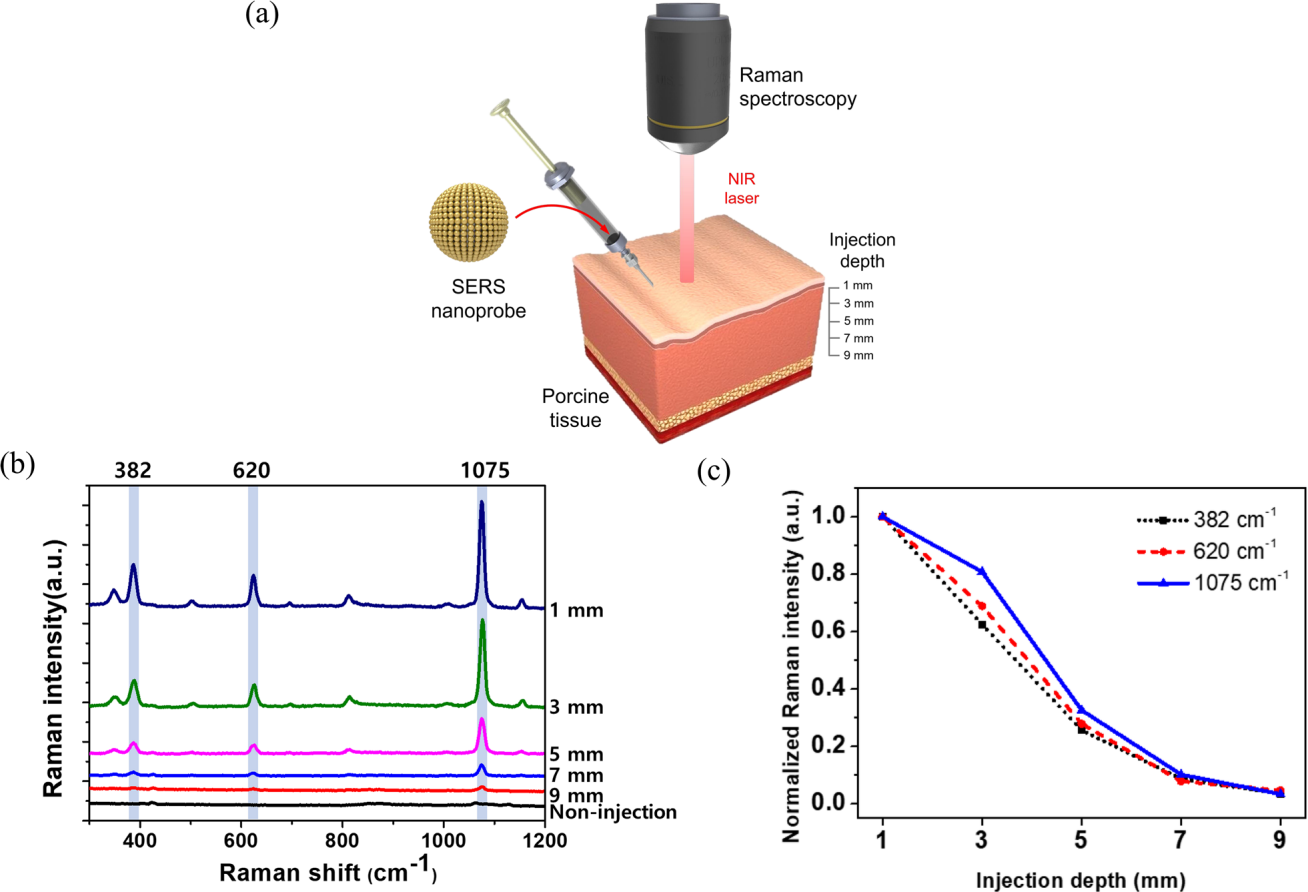
**a** Schematic illustration for the depth profile evaluation of SiO_2_@Au@Au_500_-4-FBT using the porcine tissue. **b** Raman spectra of SiO_2_@Au@Au_500_-4-FBT injected into the porcine tissue at different depths (1, 3, 5, 7, and 9 mm). **c** Correlation between normalized SERS intensities at 382, 620, and 1075 cm^−1^ for Raman spectra in **b** and the injection depth from the surface of the porcine tissue. The Raman intensity decreased as the injection depth of SiO_2_@Au@Au_500_-4-FBT increased, and was detectable up to the injection depth of 7 mm

To investigate the SERS signal depth profile of SiO_2_@Au@Au, we injected SiO_2_@Au@Au_500_-4-FBT NPs into the porcine tissue at different depths (1, 3, 5, 7, and 9 mm) and measured the SERS spectra (Fig. 4a). As the depth increased, the Raman intensity decreased (Fig. 4b). However, a measurable signal was detected up to a depth of 7 mm. For an accurate analysis, the Raman band intensities at 382, 620, and 1075 cm^−1^ were normalized to the signal intensity at a depth of 1 mm (Fig. 4c). The Raman intensity decreased as NPs were injected deeper inside the porcine tissue, and the Raman spectra distinct from that of the tissue without NP injection was observed until a depth of 7 mm. We conclude that the SiO_2_@Au@Au NPs generated a detectable SERS signal until the maximum depth of 7 mm in animal tissues. Thus, SERS detection using SiO_2_@Au@Au NPs was attempted through subcutaneous injection into animals.

**Fig. 5:**
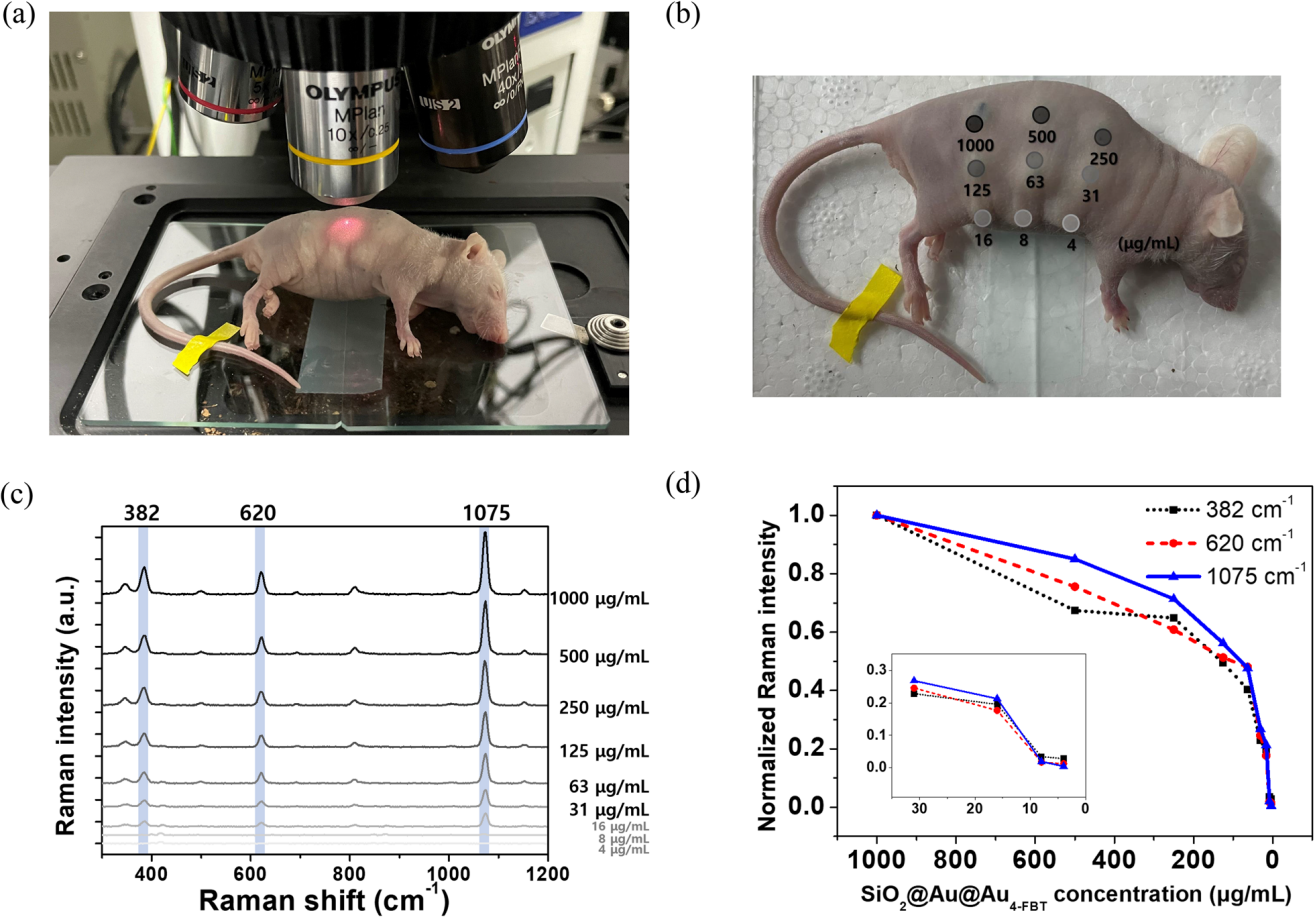
**a** Photograph of mouse injected with various concentrations of SiO_2_@Au@Au_500_-4-FBT and (**b**) the injection position. **c** Raman spectra of SiO_2_@Au@Au_500_-4-FBT injected at concentrations from 1000 to 4 μg/mL. **d** Normalized SERS intensities at 382, 620, and 1075 cm^−1^ for Raman spectra in **c**. Raman intensity-concentration curve revealed logarithmic relationship between SiO_2_@Au@Au_500_-4-FBT concentration and SERS intensity

For in vivo imaging, it is crucial to use small amounts of NPs to avoid side effects such as blood clots [46]. To determine the detectable concentration limit, various concentrations of SiO_2_@Au@Au_500_-4-FBT from 1000 to 4 μg/mL were subcutaneously injected into nude mice, and the SERS spectra were measured using a 785-nm laser (Fig. 5a, b). The Raman intensity decreased as the concentration of SiO_2_@Au@Au_500_-4-FBT NPs decreased; however, a sufficient signal was observed at a concentration of 16 μg/mL (Fig. 5c). To compare these results, the Raman bands intensities at 382, 620, and 1075 cm^−1^ were normalized to the Raman signal at 1000 μg/mL (Fig. 5d). The strong SERS signal of SiO_2_@Au@Au_500_-4-FBT allowed for the subcutaneous detection of particles even at a very low concentration (16 μg/mL), showing sufficient signal sensitivity.

### In vivo multiplex imaging potential

**Fig. 6:**
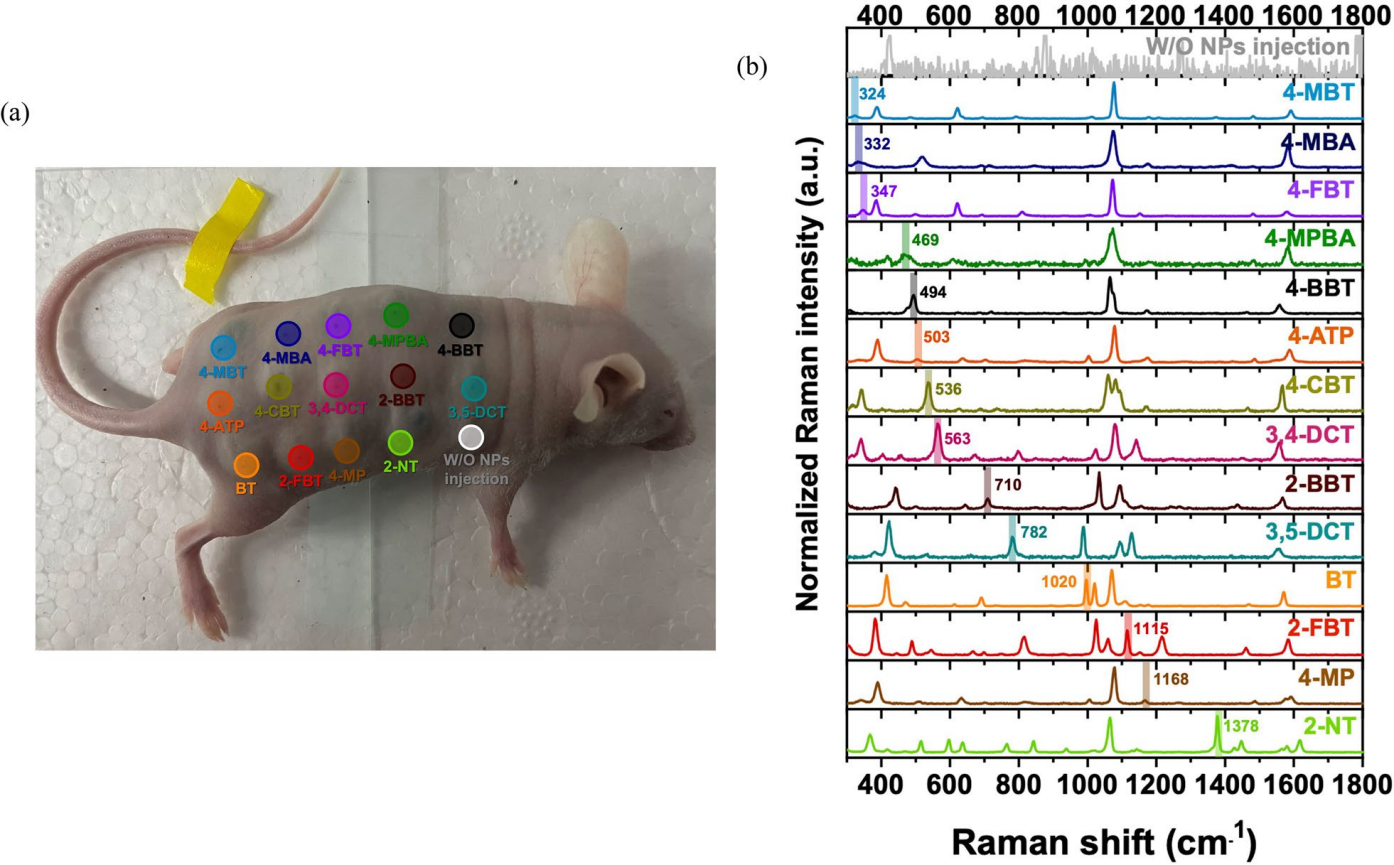
**a** Photograph of mouse injected with 14 different SiO_2_@Au@Au-RLC with 14 different Raman labeling compounds (RLCs). **b** Comparison of the 14 normalized Raman spectra of SiO_2_@Au@ Au-RLC injected into nude mouse with spectra from location without NP injection at 785-nm photoexcitation light, 2.1-mW laser power, and 10-s acquisition time. Each showed distinct Raman spectra with unique bands for label identification

To investigate the multiplex imaging potential of SiO_2_@Au@Au, 14 different RLC-treated NPs (SiO_2_@Au@Au-RLC) were prepared and injected subcutaneously into nude mice (Fig. 6a). The Raman spectra from each location were measured using a 785-nm laser. Distinct Raman spectra were obtained for the 14 types of NPs (Fig. 6b), which showed unique bands for code (label) identification (4-MBT: 324 cm^−1^; 4-MBA: 332 cm^−1^; 4-FBT: 347 cm^−1^; 4-MPBA: 469 cm^−1^; 4-BBT: 494 cm^−1^; 4-ATP: 503 cm^−1^; 4-CBT: 536 cm^−1^; 3,4-DCT: 563 cm^−1^; 2-BBT: 710 cm^−1^; 3,5-DCT: 782 cm^−1^; BT: 1020 cm^−1^; 2-FBT: 1115 cm^−1^; 4-MP: 1168 cm^−1^; and 2-NT: 1378 cm^−1^). To the best of our knowledge, the present study used the highest number of labels for NIR-active nanoprobes; the previously reported maximum number of RLCs for multiplex imaging based on SERS was 10 [3]. Thus, our SiO_2_@Au@Au NPs with multiple hotspots and narrow nanogaps exhibited high stability, allowing attachment of 14 different RLCs.

## Conclusions

SiO_2_@Au@Au NPs were prepared using the seed-mediated growth method; six SiO_2_@Au@Au NPs of different sizes were fabricated on the surface of SiO_2_ NPs by controlling the concentration of Au precursor (50, 100, 200, 300, 400, and 500 μM). With increase in concentration of Au precursor, SiO_2_@Au@Au showed stronger absorbance, particularly in the NIR region. In addition, multiple hotspots and narrow nanogaps of approximately 1 nm were obtained by increasing the concentration of Au precursor during the growth process, enabling single particle-level detection. The SERS measurement revealed the Raman signal of high intensity after 785-nm laser photoexcitation. SiO_2_@Au@Au NPs obtained using 500 μM Au precursor exhibited an average SERS EF value of 3.83 × 10^6^. SiO_2_@Au@Au NPs were successfully applied for the SERS imaging of HCT 116 cancer cells. In addition, owing to the advantages of NIR radiation and detection, the SERS signal could be measured even at a depth of 7 mm in the porcine tissue. The detectable concentration limit of NPs for subcutaneous injection was 16 μg/mL. Moreover, the multiplexing capability of the prepared SiO_2_@Au@Au was investigated by subcutaneously injecting 14 different SiO_2_@Au@Au-RLC NPs into nude mice. In this study, we fabricated highly sensitive NIR SERS nanoprobes with very strong SERS signals owing to their structure with uniformly synthesized multiple hotspots and narrow nanogaps. Along with the advantageous features of absorbing long-wavelength light and highly enhanced Raman signals, our SiO_2_@Au@Au structure can potentially be used for multiplex molecular imaging and in vivo applications.

## Supplementary Information

**Additional file 1: Fig S1**. TEM image of SiO_2_ NPs. **Fig S2.** TEM image of SiO_2_@Au used as a seed. **Fig S3.** TEM image of SiO_2_@Au@Au synthesized using 600 μM gold(III) chloride hydrate. **Fig S4.** TEM image of SiO_2_@Au synthesized by directly attaching large-sized Au NPs (10–15 nm) to aminated silica (not a growth method). **Fig S5**. SERS intensities of SiO_2_@Au@Au-4-FBT synthesized using various concentrations of gold(III) chloride hydrate determined at (a) blue visible light (wavelength, 532 nm) and (b) red visible light (wavelength, 660 nm). **Fig S6**. Cytotoxicity test using HCT 116 cells incubated with different concentrations of SiO_2_@Au@Au_500_-4-FBT. **Fig S7**. (a) Optical image, SERS mapping image, and overlay image of human colon carcinoma (HCT 116) cells incubated with 50 μg/mL SiO_2_@Au@Au_500_-4-FBT. (b) Raman intensities at different locations: outside the cell (i), on the cell surface (ii), and inside the cell (iii), corresponding to the overlay images shown in (a).

## Data Availability

All data generated or analyzed during this study are included in this published article and its Additional files.

## Abbreviations

AA: Ascorbic acid
APTS: (3-Aminopropyl)triethoxysilane
ATCC: American Type Culture Collection
BT: Benzenethiol
DW: Deionized water
EtOH: Ethanol
FBS: Fetal bovine serum
HAuCl_4_: Gold(III) chloride trihydrate
NaOH: Sodium hydroxide
NH_4_OH: Aqueous ammonium hydroxide
NIR: Near-infrared
NPs: Nanoparticles
PBS: Phosphate-buffered saline
PTT: Photothermal therapy
PVP: Polyvinylpyrrolidone
RLCs: Raman labeling compounds
SERS: Surface-enhanced Raman spectroscopy
SDS: Sodium dodecyl sulphate
SiO_2_: Silica
SiO_2_@Au@Au: Au-assembled silica nanoparticle
TEOS: Tetraethyl orthosilicate
THPC: Tetrakis(hydroxymethyl)-phosphonium chloride
2-BBT: 2-Bromobenzenethiol
2-FBT: 2- Fluorobenzenethiol
2-NT: 2-Naphthalenethiol
3,4-DCT: 3,4-Dichlorobenzenethiol
3,5-DCT: 3,5-Dichlorobenzenethiol
4-ATP: 4-Aminothiophenol
4-BBT: 4-Bromobenzenethiol
4-CBT: 4-Chlorobenzenethiol
4-FBT: 4-Fluorobenzenethiol
4-MBA: 4-Mercaptobenzoic acid
4-MBT: 4-Methylbenzenethiol
4-MP: 4-Mercaptophenol
4-MPBA: 4-Mercaptophenyl boronic acid
