# Supplementary material for: Highly sensitive near-infrared SERS nanoprobes for in vivo imaging using gold-assembled silica nanoparticles with controllable nanogaps
Source: J Nanobiotechnology. 2022 Mar 12;20:130. doi: 10.1186/s12951-022-01327-7 (PMC8917682; doi:10.1186/s12951-022-01327-7)
Supplement: Supplementary file 1 — Additional file 1: Fig S1. TEM image of SiO2 NPs. Fig S2. TEM image of SiO2@Au used as a seed. Fig S3. TEM image of SiO2@Au@Au synthesized using 600 μM gold(III) chloride hydrate. Fig S4. TEM image of SiO2@Au synthesized by directly attaching large-sized Au NPs (10–15 nm) to aminated silica (not a growth method). Fig S5. SERS intensities of SiO2@Au@Au-4-FBT synthesized using various concentrations of gold(III) chloride hydrate determined at (a) blue visible light (wavelength, 532 nm) and (b) red visible light (wavelength, 660 nm). Fig S6. Cytotoxicity test using HCT 116 cells incubated with different concentrations of SiO2@Au@Au500-4-FBT. Fig S7. (a) Optical image, SERS mapping image, and overlay image of human colon carcinoma (HCT 116) cells incubated with 50 μg/mL SiO2@Au@Au500-4-FBT. (b) Raman intensities at different locations: outside the cell (i), on the cell surface (ii), and inside the cell (iii), corresponding to the overlay images shown in (a). [file 12951_2022_1327_MOESM1_ESM.docx]

**Highly Sensitive Near-Infrared SERS Nanoprobes for In Vivo Imaging using Gold-assembled Silica Nanoparticles with Controllable Nanogaps**

Sungje Bock^1, ‡^, Yun-Sik Choi^2, ‡^, Minhee Kim^1,‡^, Yewon Yun^1^, Xuan-Hung Pham^1^, Jaehi Kim^1^, Bomi Seong^1^, Wooyeon Kim^1^, Ahla Jo^1^, Kyeong-Min Ham^1^, Sung Gun Lee^2^, Sang Hun Lee^3^, Homan Kang^4^, Hak Soo Choi^4^, Dae Hong Jeong^2^, Hyejin Chang^5,*^, Dong-Eun Kim^1,*^, and Bong-Hyun Jun^1,*^

‡ These authors contributed equally to this work.

*Correspondence: Hyejin Chang, hjchang@kangwon.ac.kr; Dong-Eun Kim, kimde@konkuk.ac.kr; Bong-Hyun Jun, bjun@konukuk.ac.kr

^1^Department of Bioscience and Biotechnology, Konkuk University, Seoul 05029, South Korea

^5^Division of Science Education, Kangwon National University, Chuncheon 24341, south Korea

Full list of author information is available at the end of the article


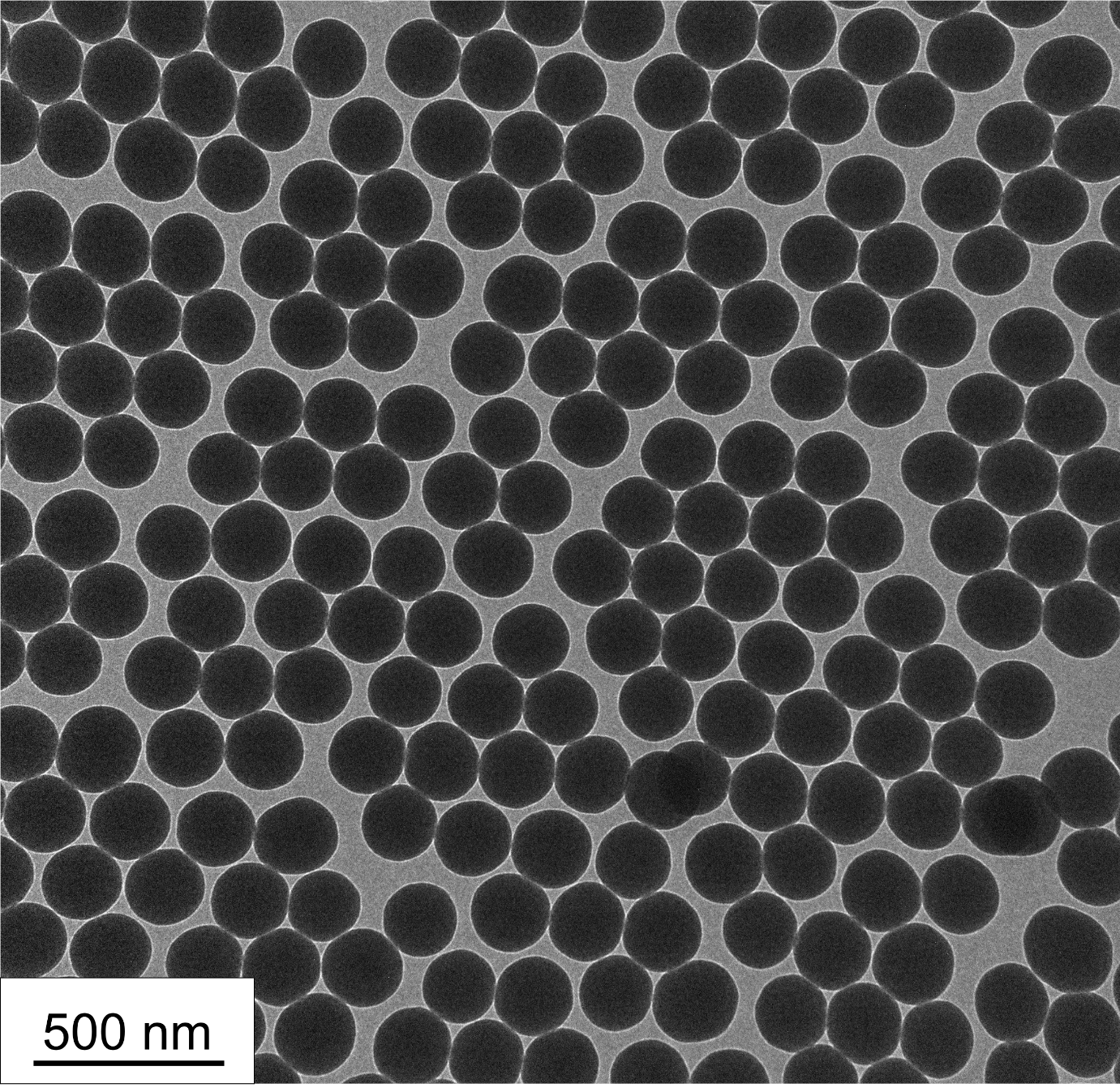


**Figure S1**. TEM image of SiO_2_ NPs.


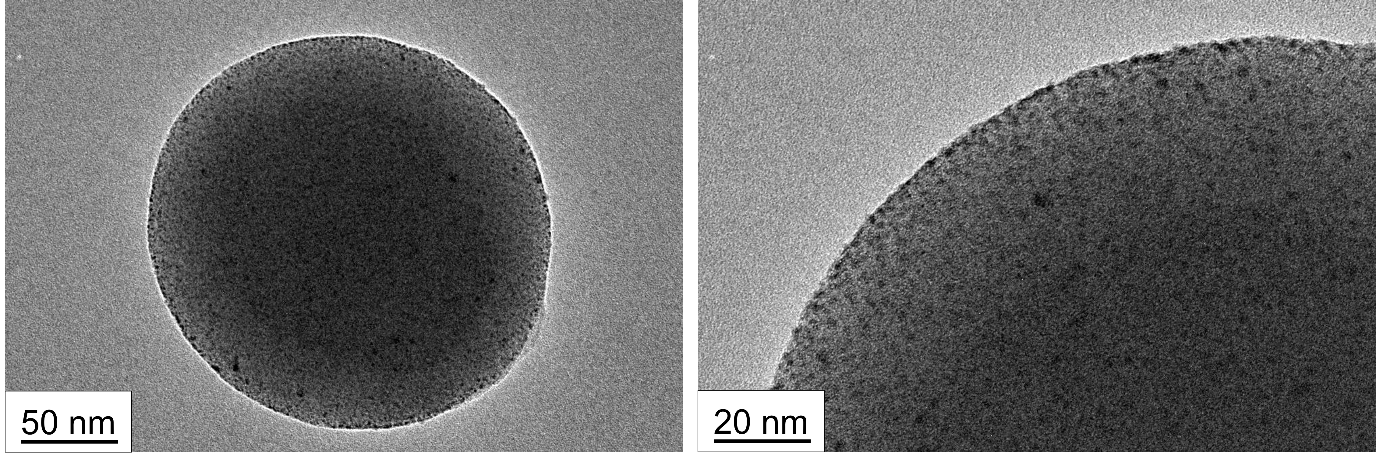


**Figure S2**. TEM images of SiO_2_@Au used as a seed.


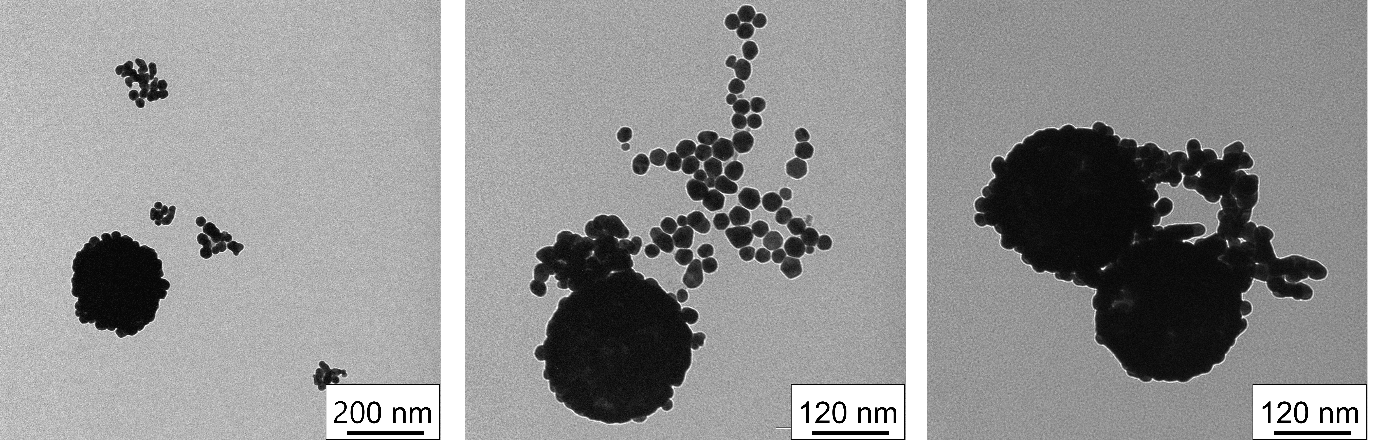


**Figure S3**. TEM images of SiO_2_@Au@Au synthesized with 600 μM gold(III) chloride hydrate.


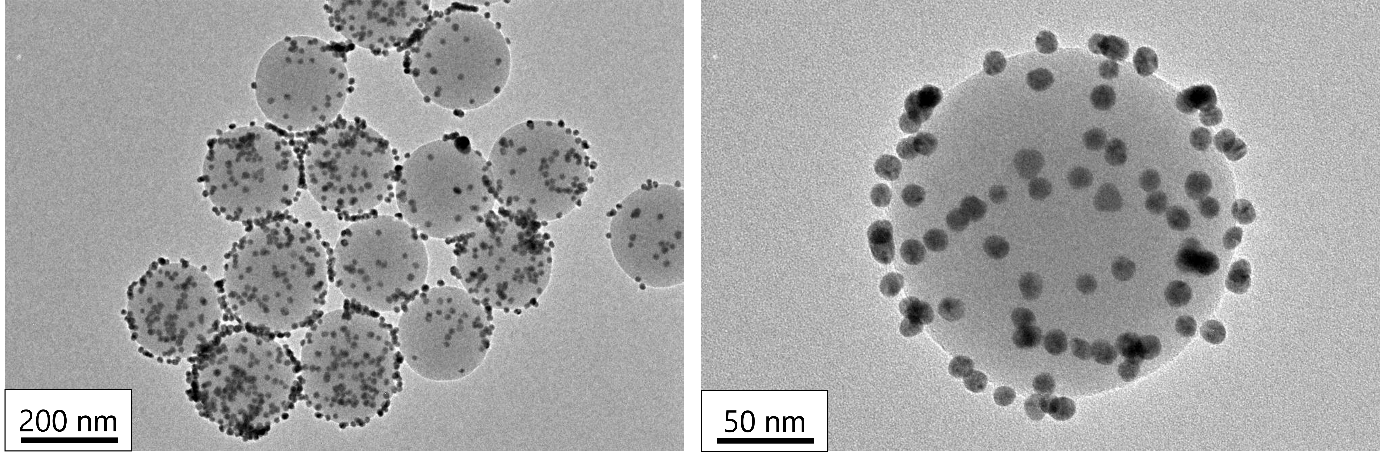


**Figure S4**. TEM images of SiO_2_@Au synthesized by directly attaching large Au NPs (10–15 nm) to aminated silica (not a growth method).


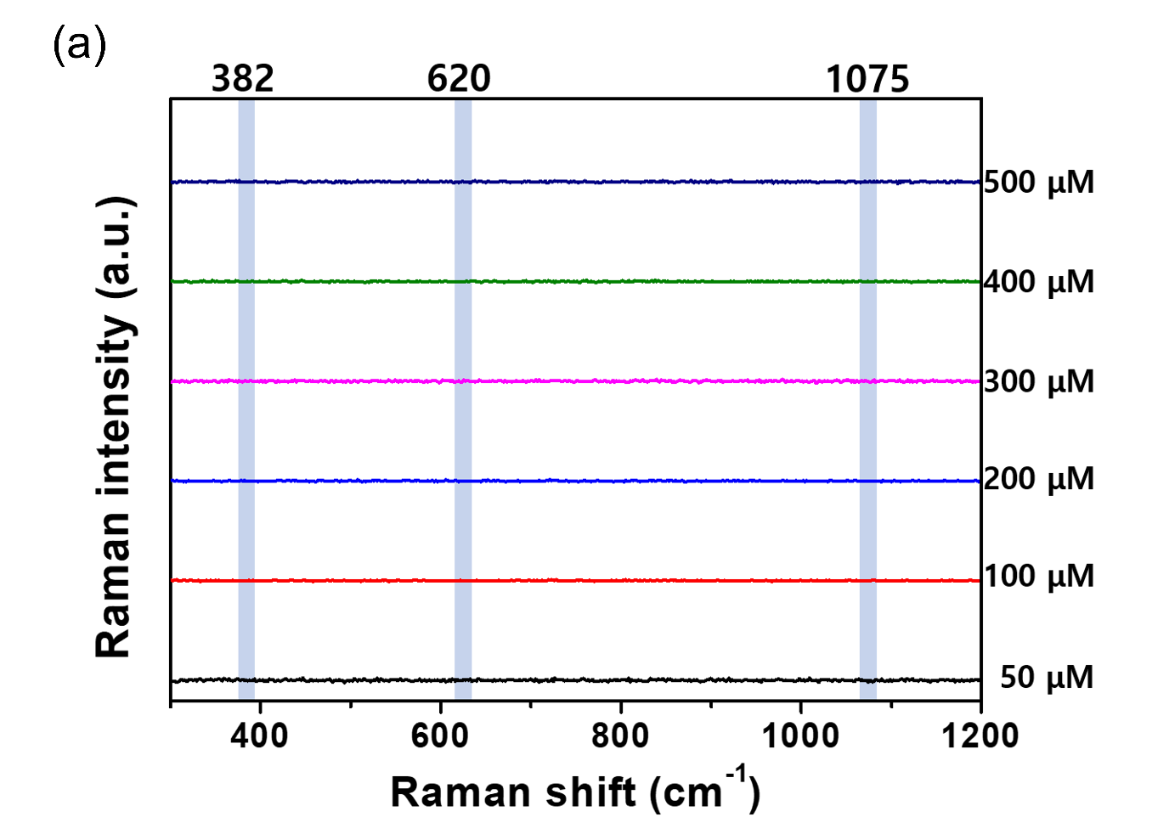

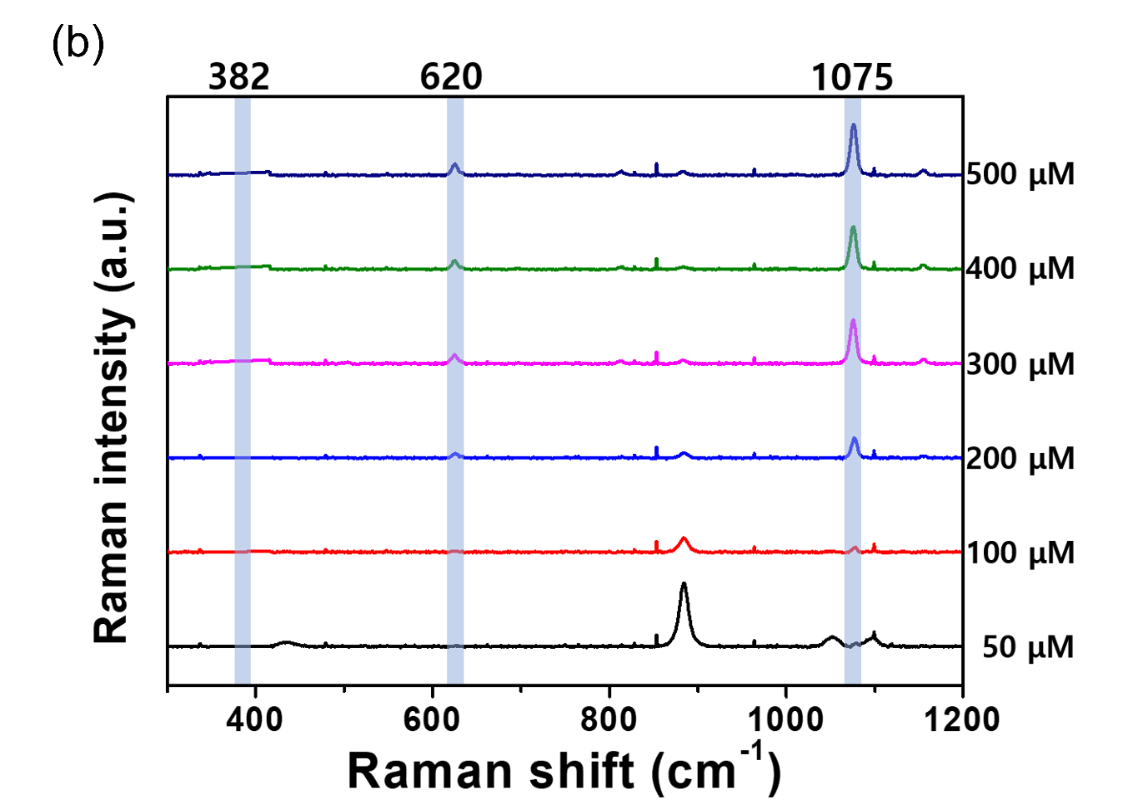


**Figure S5**. SERS intensities of SiO_2_@Au@Au-4-FBT with various concentrations of gold(III) chloride hydrate determined using (a) blue visible light (wavelength, 532 nm) and (b) red visible light (wavelength, 660 nm).


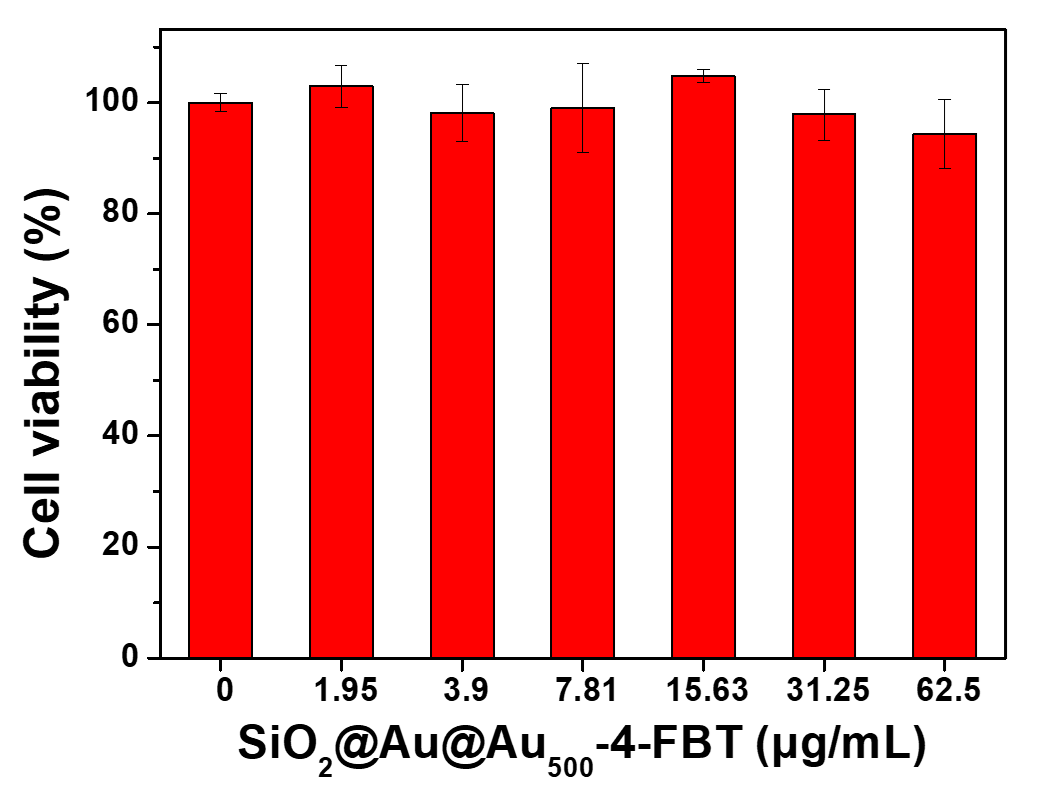


**Figure S6**. Cytotoxicity test using HCT 116 cells incubated with different concentrations of SiO_2_@Au@Au_500_-4-FBT.


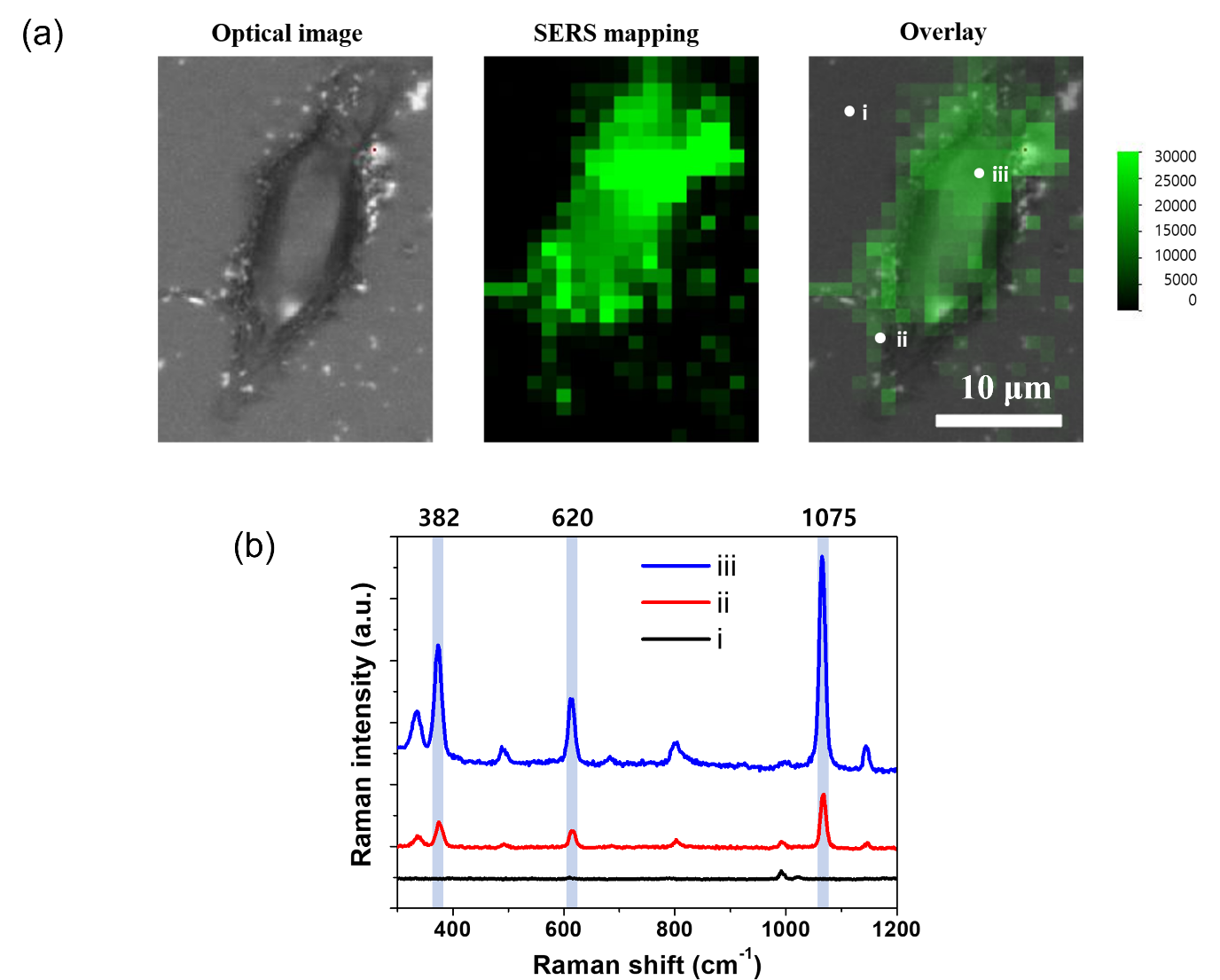


**Figure S7**. (a) Optical image, SERS mapping image, and overlay image toward human colon carcinoma (HCT 116) cells incubated with 50 μg/mL SiO_2_@Au@Au_500_-4-FBT. (b) Raman intensities at indicated points, location outside the cell (i), on the cell surface (ii), and inside the cell (iii), corresponding to the overlay images shown in (a).
